# Human Cytomegalovirus pUL79 Is an Elongation Factor of RNA Polymerase II for Viral Gene Transcription

**DOI:** 10.1371/journal.ppat.1004350

**Published:** 2014-08-28

**Authors:** Yi-Chieh Perng, Jessica A. Campbell, Deborah J. Lenschow, Dong Yu

**Affiliations:** 1 Department of Molecular Microbiology, Washington University School of Medicine, Saint Louis, Missouri, United States of America; 2 Department of Medicine, Department of Pathology and Immunology, Washington University School of Medicine, Saint Louis, Missouri, United States of America; Oregon Health and Science University, United States of America

## Abstract

In this study, we have identified a unique mechanism in which human cytomegalovirus (HCMV) protein pUL79 acts as an elongation factor to direct cellular RNA polymerase II for viral transcription during late times of infection. We and others previously reported that pUL79 and its homologues are required for viral transcript accumulation after viral DNA synthesis. We hypothesized that pUL79 represented a unique mechanism to regulate viral transcription at late times during HCMV infection. To test this hypothesis, we analyzed the proteome associated with pUL79 during virus infection by mass spectrometry. We identified both cellular transcriptional factors, including multiple RNA polymerase II (RNAP II) subunits, and novel viral transactivators, including pUL87 and pUL95, as protein binding partners of pUL79. Co-immunoprecipitation (co-IP) followed by immunoblot analysis confirmed the pUL79-RNAP II interaction, and this interaction was independent of any other viral proteins. Using a recombinant HCMV virus where pUL79 protein is conditionally regulated by a protein destabilization domain *dd*FKBP, we showed that this interaction did not alter the total levels of RNAP II or its recruitment to viral late promoters. Furthermore, pUL79 did not alter the phosphorylation profiles of the RNAP II C-terminal domain, which was critical for transcriptional regulation. Rather, a nuclear run-on assay indicated that, in the absence of pUL79, RNAP II failed to elongate and stalled on the viral DNA. pUL79-dependent RNAP II elongation was required for transcription from all three kinetic classes of viral genes (i.e. immediate-early, early, and late) at late times during virus infection. In contrast, host gene transcription during HCMV infection was independent of pUL79. In summary, we have identified a novel viral mechanism by which pUL79, and potentially other viral factors, regulates the rate of RNAP II transcription machinery on viral transcription during late stages of HCMV infection.

## Author Summary

In this study, we report a novel mechanism used by human cytomegalovirus (HCMV) to regulate the elongation rate of RNA polymerase II (RNAP II) to facilitate viral transcription during late stages of infection. Recently, we and others have identified several viral factors that regulate gene expression during late infection. These factors are functionally conserved among beta- and gamma- herpesviruses, suggesting a unique transcriptional regulation shared by viruses of these two subfamilies. However, the mechanism remains elusive. Here we show that HCMV pUL79, one of these factors, interacts with RNAP II as well as other viral factors involved in late gene expression. We have started to elucidate the nature of the pUL79-RNAP II interaction, finding that pUL79 does not alter the protein levels of RNAP II or its recruitment to viral promoters. However, during late times of infection, pUL79 helps RNAP II efficiently elongate along the viral DNA template to transcribe HCMV genes. Host genes are not regulated by this pUL79-mediated mechanism. Therefore, our study discovers a previously uncharacterized mechanism where RNAP II activity is modulated by viral factor pUL79, and potentially other viral factors as well, for coordinated viral transcription.

## Introduction

HCMV is a prototypical beta-herpesvirus and a ubiquitous pathogen in the human population. Upon primary infection, HCMV establishes a lifelong persistent and latent/recurrent infection in a host [1]. Even though HCMV infection is usually asymptomatic, it acts as an opportunistic pathogen and is a major cause of morbidity and mortality in immunocompromised individuals, including transplant recipients and AIDS/HIV patients [2]. Importantly, HCMV is the leading infectious cause of birth defects in newborns [3]. Furthermore, there is evidence for HCMV to act as a risk factor in the development of vascular diseases, such as atherosclerosis, transplant vascular sclerosis, and coronary restenosis after angioplasty surgery [4]–[10]. Finally, HCMV has also been suggested to be relevant to multiple forms of human cancers, where it may have a potential contribution to oncogenic transformation, onco-modulation, and tumor cell immune evasion [11]–[14].

During lytic infection, HCMV genes are expressed in a highly ordered temporal cascade (reviewed in [15]–[18]). Viral transcripts accumulate with three kinetic classes, namely immediate-early, early, and late. The HCMV major IE (MIE) genes UL123 (IE1) and UL122 (IE2) play critical roles in predisposing the cellular environment to infection and also act as transactivators to induce early gene transcription. Many early genes encode proteins required for viral DNA synthesis [19]–[21]. The transcript accumulation of early genes is independent of viral DNA synthesis; however, the continued accumulation of a subset of genes (i.e., early-late) is enhanced by the onset of viral DNA synthesis [22]. Following viral DNA replication, late viral genes, which mainly encode structural proteins, start to transcribe and ultimately lead to the assembly and release of infectious particles. Previous studies have shown that the activation of both beta- and gamma-herpesvirus late gene promoters is dependent on the origin of viral DNA synthesis (OriLyt) *in cis* [23]–[25]. This further supports the notion that late gene transcription is tightly coupled to viral DNA synthesis. However, whether viral late gene expression is subjected to additional viral regulation remains poorly defined.

In many DNA viruses, viral gene expression during productive infection is also temporally regulated and can be divided into early and late phases separated by viral genome replication. However, the mechanisms of late gene expression are diverse. Simian virus 40 (SV40) requires viral DNA replication *in trans* to relieve the repression of viral late promoters [26], [27], and the viral large T antigen also plays a critical role to activate the late promoters [28], [29]. Viral late gene expression during papillomavirus infection is tightly associated with keratinocyte differentiation and mediated in part by alternative mRNA splicing [30]. For adenoviruses, activation of late gene expression requires both *cis* elements of viral DNA replication [31], [32] and *trans* acting factors to titrate an inhibitory factor during viral DNA synthesis [33]. For herpesviruses, viral late gene expression has been studied extensively with herpes simplex virus (HSV). In HSV, viral DNA replication is required in *cis* for activity of late promoters [34], [35]. HSV proteins, including ICP4, ICP8, and ICP27, facilitate the assembly of transcription preinitiation complexes [36], [37], and are required for efficient expression of late genes by interacting with the general transcription machinery [38]–[40]. However, the regulatory activities of these viral proteins in late gene expression are not well conserved in beta- and gamma-herpesviruses.

Recently, we and others have demonstrated that HCMV encodes five essential proteins, UL79, UL87, UL91, UL92, and UL95, which are required for the expression of viral late genes after viral DNA synthesis [41]–[43]. Murine cytomegalovirus (MCMV) M79 and M92, homologs of HCMV UL79 and UL92, respectively, are also required for late gene expression [44], [45]. Homologs of UL79, UL87, UL91, UL92, and UL95 are found in murine gammaherpesvirus 68 (MHV-68) (ORF18, ORF24, ORF30, ORF31, and ORF34, respectively), which have been shown to have similar functions [46]–[49]. Epstein-Barr virus (EBV) BcRF1, a UL87 homolog, is a novel viral TATA-box binding protein with greater specificity for a non-classical TATA-box sequence [50], [51]. Intriguingly, these factors are conserved only in beta- and gamma-herpesviruses and have no known homologues in herpes simplex virus (HSV) [18], [41], suggesting a unique viral regulatory mechanism shared by these two herpesviral subfamilies. However, the underlying mechanisms of how these viral factors regulate late gene expression are incompletely understood.

During cytomegalovirus infection, viral genes are transcribed by cellular RNA polymerase II (RNAP II). Its largest subunit Rpb1 has a carboxy terminal domain (CTD) containing 52 repeats of a heptapeptide (Tyr^1^-Ser^2^-Pro^3^-Thr^4^-Ser^5^-Pro^6^-Ser^7^) [52]. The CTD acts as a scaffold to interact with other transcription factors and coordinate transcription with other processes, such as mRNA maturation and chromatin modification [53], [54]. This activity is tightly regulated by the phosphorylation status of the CTD [55], [56]. Unphosphorylated RNAP II is recruited to preinitiation complexes (PIC) [57]. Once bound to a promoter, CTD Ser^5^ is phosphorylated by cdk7 to release RNAP II from the PIC [58] and also promote the recruitment of capping/splicing factors and histone modification complexes [53]. RNAP II then proceeds to intrinsic pausing sites where it is halted by negative elongation factors (NELFs). The onset of productive elongation requires the positive transcription elongation factor P-TEFb composed of cdk9 and cyclin T, which phosphorylates CTD Ser^2^ [59]. At the 3′ end of the coding region, phosphatases Ssu72 and Fcp1 dephosphorylate the CTD. RNAP II dissociates from the DNA template and is recycled as an unphosphorylated, initiation-competent form for another round of transcription [60], [61].

HCMV utilizes RNAP II and the accompanying host machinery for transcription of viral genes. During early times of viral infection, RNAP II and other transcription machinery are recruited to early replication sites to drive viral IE and early gene expression [62]. The protein levels of RNAP II, including hyper-phosphorylated forms, increase as infection progresses [62], [63]. Treatment of infected cells with cdk inhibitors inhibits viral gene expression as well as viral replication [64]. During late stages of viral infection, cdk kinase and RNAP II-associated transcriptional machinery proteins continue to accumulate and relocate into the peri-replication center [65]. However, how RNAP II transcription machinery remains active on viral loci during late infection requires further investigation.

In this study, we dissected the mechanism of HCMV late gene expression by investigating the proteins that are associated with late transcription regulator pUL79 during HCMV infection. We found that pUL79 interacted with a panel of viral and host proteins, including RNAP II, other novel late transcription regulators pUL87 and pUL95, as well as components of the viral DNA replication complex. We delineated the pUL79-RNAP II interaction and found that pUL79 bound to RNAP II in the nucleus independent of additional viral factors. Mechanistically, pUL79 did not alter RNAP II protein levels or the phosphorylation profile of its CTD. Instead, in the absence of pUL79, RNAP II stalled on viral DNA loci, including those of viral immediate-early, early, and late genes, but not those of host genes, during late times of infection. This resulted in a significantly diminished elongation rate of RNAP II-driven transcription on viral loci. We conclude that during late times of infection HCMV induces the formation of unique transcriptional machinery in which pUL79 acts as an elongation factor to specifically drive RNAP II-mediated transcription on the viral genome.

## Results

### Identification of pUL79-interacting proteins

**Figure 1:**
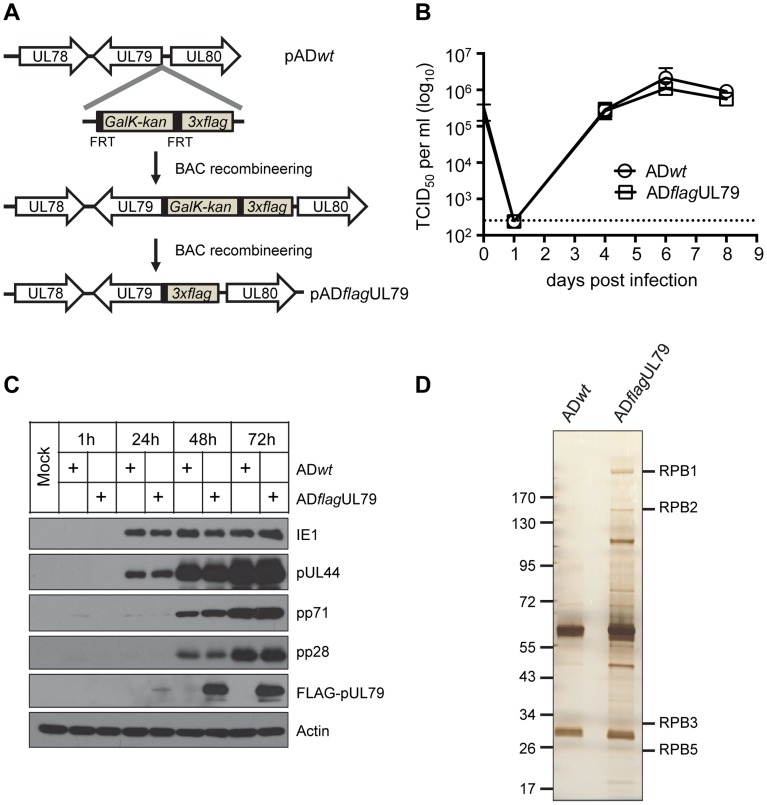
Identification of pUL79 interacting proteins. (A) Schematic diagram for creating pAD*flag*UL79, the recombinant HCMV BAC clone used to produce virus AD*flag*UL79. A cassette that contained a 3×FLAG tag followed by the FRT-bracketed GalK/kanamycin dual selection marker was amplified by PCR and recombined into the wildtype HCMV BAC clone (pAD*wt*) at the 5′ terminus of the UL79 coding sequence. The selection marker was then removed by Flp/FRT recombination. The final clone, pAD*flag*UL79, carried the UL79 coding sequence tagged at its 5′ terminus with 3×FLAG. (B) Single step viral growth analysis. HFF cells were infected with HCMV recombinant virus AD*flag*UL79 (derived from pAD*flag*UL79) or AD*wt* (derived from pAD*wt*) at an MOI of 3. Infected culture supernatants were collected at indicated days post infection and virus titers were determined by TCID_50_ assay. The mean virus titers were derived from two independent experiments and two technical replicates. Standard deviations are presented. The detection limit is indicated by the dashed line. (C) Viral protein expression profile. HFFs were infected as described in (B), and harvested at indicated times post infection. Accumulations of host and viral proteins were determined by immunoblot analysis. FLAG-tagged pUL79 was detected by an anti-FLAG antibody. Actin was used as a loading control. Representative results from three independent experiments are shown. (D) Polyacrylamide gel electrophoresis to resolve pUL79 protein complexes. HFFs were infected as described in (B), and at 72 hpi, cell lysates were prepared for immunoprecipitation using an anti-FLAG antibody. Immunoprecipitated proteins were resolved on a gradient polyacrylamide gel and silver stained. Protein bands containing RNAP II subunits identified by mass spectrometry are indicated. Molecular size markers (in kilodaltons) are shown.

To investigate proteins associated with pUL79, we first generated a recombinant HCMV in which the UL79 coding sequence was tagged with the 3×FLAG sequence (AD*flag*UL79) so that protein complexes containing pUL79 in infected cell lysate could be isolated by immunoprecipitation (IP) with an anti-FLAG antibody (Fig. 1A). Both growth and protein expression profile (Figs. 1B–1C) of AD*flag*UL79 was indistinguishable from those of wildtype AD169 strain (AD*wt*) in human foreskin fibroblasts cells (HFFs). These results indicate that the addition of 3×FLAG tag to the N-terminus of the UL79 coding sequence does not compromise the function of pUL79.

To identify proteins that interacted with FLAG-pUL79, lysates from HFF cells infected with virus AD*flag*UL79 or AD*wt* (negative control) were collected at 72 hours post infection (hpi) and immunoprecipitated with the anti-FLAG antibody. Immunoprecipitated proteins were resolved by SDS-PAGE and visualized by silver staining (Fig. 1D). Protein bands unique to AD*flag*UL79 were extracted and their identities were determined by mass spectrometry. For the negative control, we also extracted gel bands from the AD*wt* sample with migrating positions corresponding to those of AD*flag*UL79-specific protein bands as negative controls for mass-spectrometry analysis. The full set of proteins that were identified by this approach and unique to AD*flag*UL79 is listed in Table 1.

**Table 1:** pUL79 protein partners identified by mass spectrometry.

Protein ID	Description	Size (kDa)	Expectation^a^	Peptide count
**HCMV transactivators**				
B8YEB2	pUL87	104.7	0	44
B8YEB9	pUL95	57.2	9.4×10E-37	26
A8T7F6	pUL79	33.8	1.5×10E-40	12
**HCMV DNA synthesis**				
Q69214	UL112/113 (pp34)	28.3	2.7×10E-14	7
D2K4M1	UL112 (pp84)	70.2	3.2×10E-15	6
D2K5E9	pUL44 (DNA pol. processivity factor)	46.2	8.0×10E-6	2
C8CFZ3	IRS1 (tegument protein)	91.7	9.6×10E-3	1
**Other HCMV proteins**				
D2K3R0	pUL104 (capsid portal protein)	78.4	2.5×10E-35	10
D2K4X8	pUL85 (capsid triplex subunit)	34.5	2.8×10E-16	7
D2K4H6	pUL150 (nuclear egress protein)	43.1	1.1×10E-05	2
Q1KQ04	pUL49	63.8	0.01	1
**Cellular RNA polymerase II**				
RPB2_Human	Subunit Rpb2	133.8	8.8×10E-101	29
RPB1_Human	Subunit Rpb1	217	2.6×10E-81	27
RPB3_Human	Subunit Rpb3	31.4	1.1×10E-20	6
RPB5_Human	Subunit Rpb5	24.5	6.8×10E-07	2
**Ribosome biogenesis**				
RL7A_Human	60S ribosomal protein L7a	29.9	4.6×10E-07	3
RL21_Human	60S ribosomal protein L21a	18.5	2.1×10E-11	3
RS2_Human	40S ribosomal protein RPS2	31.3	6.7×10E-05	3
RS3A_Human	40S ribosomal protein RPS3a	29.9	4.4×10E-04	3
RL23_Human	60S ribosomal protein RPL23	14.8	3.9×10E-04	2
RL23A_Human	60S ribosomal protein RPL23a	17.6	3.6×10E-04	1
RL24_Human	60S ribosomal protein L24	17.7	8.8×10E-01	1
RS26L_Human	40S ribosomal protein S26-like 1	12.9	9.8×10E-03	1
RS27A_Human	Ubiquitin-40S ribosomal protein S27a	17.9	0.23	1
**Other cellular proteins**				
ACTB_Human	Actin, cytoplasmic 1	41.9	4.6×10E-51	15
ACTN1_Human	Alpha-actinin-1	102.9	8.4×10E-10	3
EF1A1_Human	Elongation factor 1-alpha 1	50.1	4.8×10E-3	3
ANXA5_Human	Annexin A5	35.9	9.7×10E-3	3
H2A1B_Human	Histone H2A type 1-B/E	14.12	0.15	3
RECQ4_Human	ATP-dependent DNA helicase Q4	132.9	0.25	1

a Expectation value for peptide match (i.e. the number of times expected to obtain an equal or higher score, purely by chance). A lower value indicates a higher likelihood of the interaction.

These pUL79-interacting proteins could be categorized into several functional groups. Most notably, four out of twelve core subunits of human RNA polymerase II (RNAP II), namely Rpb1, Rpb2, Rpb3, and Rpb5, were identified (Table 1). Rpb1 is the largest subunit of RNAP II and its C-terminal domain (CTD) plays a critical role in transcription regulation by interacting with various transcriptional factors. Second, several viral proteins that are conserved among beta- and gamma- herpesviruses, including pUL87, pUL95, pUL49, and pUL92, were found in the pUL79-protein complexes. pUL87 and pUL95 (shown in Table 1), together with pUL79, are required for viral late gene expression and are reported to be recruited to the viral pre-replication complexes [42], [43]. pUL92, another HCMV protein required for viral late gene expression, was also identified in this mass spectrometry analysis as a pUL79-interacting protein and has been reported as such in a separate study [44]. These data together suggest that pUL79 interacts with other viral regulatory proteins involved in late gene expression during HCMV infection. Third, proteins involved in viral DNA synthesis or shown to be associated with viral lytic origin of replication (OriLyt) [66], including pUL44, pIRS1, and pUL112/113, were also found in pUL79 protein complexes. Copurification of pUL79 and viral DNA replication factors suggests that pUL79 may have a role in coordinating viral DNA synthesis and late gene expression. Finally, several cellular proteins involved in protein translation, such as ribosomal protein subunits and elongation factor 1-alpha1, were co-purified with pUL79.

In this study, we focused on the interaction between pUL79 and RNAP II subunits. As RNAP II transcribes viral genes during infection, we hypothesized that pUL79 interacts with RNAP II to modify and promote its activity for viral transcription during late stages of infection.

### pUL79 interacts with the RNAP II complex

**Figure 2:**
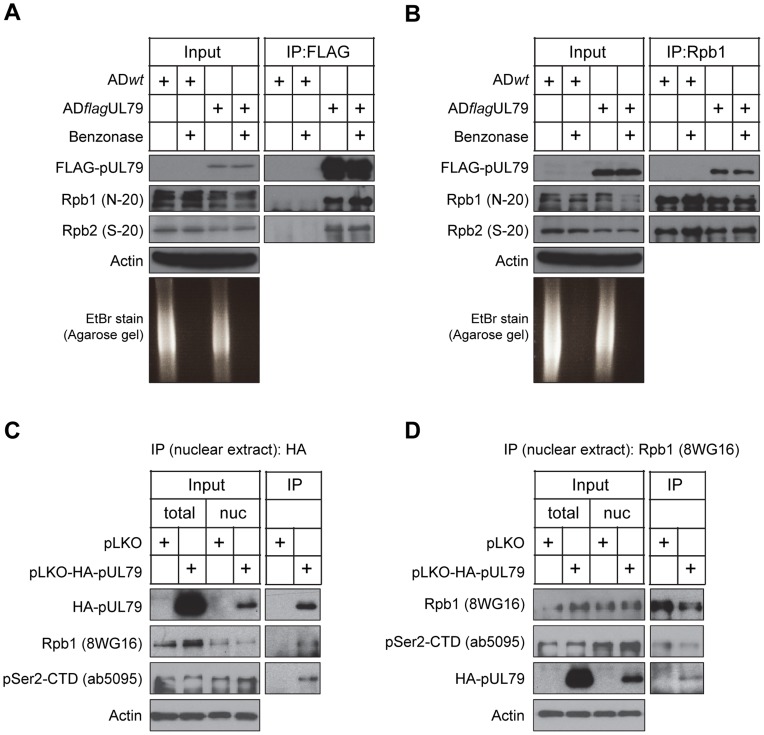
pUL79 interacts with the RNAP II protein complex. In (A–B), HFFs were infected as described in Fig. 1, and at 72 hpi cell lysates were immunoprecipitated using either an anti-FLAG antibody (A) or anti-Rpb1 antibody N-20 (B). Immunoprecipitated proteins and lysate inputs were analyzed by immunoblotting. To examine the efficiency of nuclease digestion, the immunoprecipitated samples were also analyzed on an ethidium bromide (EtBr)-stained agarose gel. In (C–D), nuclear lysates from HEK-293T cells transiently expressing HA-tagged pUL79 or empty vector control were prepared at 72 hours post transfection. Lysates were immunoprecipitated using either an anti-HA antibody (C) or anti-Rpb1 antibody 8WG16 (D). Immunoprecipitated proteins and lysate inputs were analyzed by immunoblotting. The clone names of antibodies used in immunoblot analysis are shown. Representative results from three independent experiments are presented.

To further investigate the association of the RNAP II complex with pUL79, we first validated this interaction by immunoprecipitation analysis. HFFs were infected with either AD*flag*UL79 or AD*wt* (negative control), cell lysates were collected at 72 hpi, and proteins were immunoprecipitated by using antibodies against RNAP II or FLAG, followed by immunoblot analysis (Fig. 2). For the cells infected with AD*flag*UL79, two RNAP II subunits, Rpb1 and Rpb2, were co-immunoprecipitated with FLAG-pUL79 but were not co-immunoprecipitated from AD*wt*-infected samples (Fig. 2A). In a reciprocal experiment, an anti-Rpb1 antibody co-immunoprecipitated not only the RNAP II complex (indicated by Rpb1 and Rpb2) in both AD*flag*UL79- and AD*wt*- infected samples, but also FLAG-pUL79 in AD*flag*UL79-infected samples (Fig. 2B). Taken together, these results indicate that pUL79 is associated with the RNAP II complex during viral infection.

The RNAP II complex binds to both DNA and RNA fragments. It is possible that the observed interaction of pUL79 with RNAP II is indirect, and is instead the result of the association of both proteins with the same DNA or RNA fragment. To determine if nucleic acids are required for the pUL79-RNAP II interaction, cell lysates were treated with a nonspecific nuclease (Benzonase) prior to immunoprecipitation [67]. Benzonase treatment was effective, reducing RNA/DNA to undetectable levels in ethidium bromide-stained agarose gel electrophoresis analysis (Fig. 2A and Fig. 2B). In the presence of nuclease, pUL79, Rpb1, and Rpb2 remained co-immunoprecipitated in AD*flag*UL79-infected lysates (Fig. 2A and 2B). Taken together, these results indicate that pUL79 and RNAP II associate with one another, and that this association is not mediated by nucleic acids.

We then sought to determine whether the pUL79-RNAP II interaction could form independent of additional viral factors. To achieve this, we transfected HEK-293T cells with a plasmid expressing HA-tagged pUL79 or an empty vector plasmid. pUL79 contains a PY-nuclear localization signal directing it into the nucleus [68] and is located in viral replication compartments during infection [42], [43]. Therefore, we extracted nuclear lysates of transfected cells, and performed co-immunoprecipitation analysis to examine the pUL79-RNAP II interaction using either an anti-HA antibody or anti-Rpb1 antibody in the presence of nuclease. As anticipated, HA-pUL79 was present in the nuclear extracts (Fig. 2C and 2D). Anti-HA antibody immunoprecipitated HA-pUL79 together with Rpb1, particularly the Rpb1 CTD phosphorylated at Serine 2 (pSer2-CTD) (Fig. 2C). As pSer2-CTD is a marker of RNAP II undergoing transcriptional elongation, this result suggests that pUL79 may interact with RNAP II during the transcription cycle to modulate its elongation. Reciprocal co-immunoprecipitation using an anti-Rpb1 antibody further confirmed the association of RNAP II with pUL79 (Fig. 2D). Together, these results indicate that pUL79 can interact with RNAP II independent of other viral factors. The presence of pSer2-CTD in the pUL79-RNAP II complex also suggests that pUL79 may regulate the elongation activity of RNAP II.

### pUL79 does not alter protein accumulations of RNAP II

A previous study found that HCMV promotes the accumulation of RNAP II at late times during infection [63]. Various isoforms of phosphorylated RNAP II, including pSer2-CTD and pSer5-CTD (i.e. CTD phosphorylated at Serine 5, a hallmark of successful transcription initiation) also accumulate at these late times [62], [63], [69]. However, the mechanism of how HCMV regulates these RNAP II-mediated transcriptional events is not clear.

**Figure 3:**
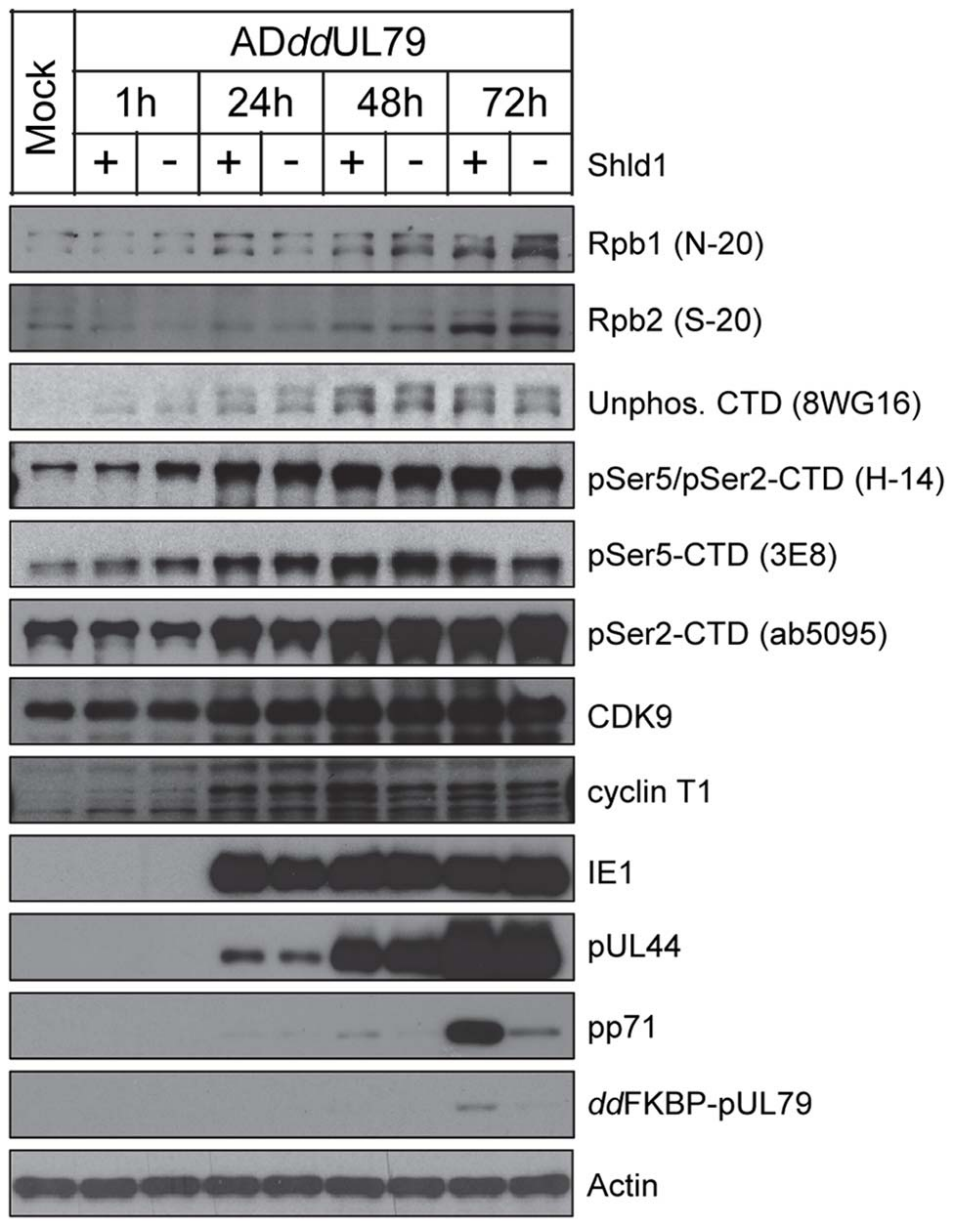
pUL79 does not alter protein accumulations of RNAP II. HFFs were infected with AD*dd*UL79 at an MOI of 3 in the presence or absence of 1 µM Shield-1 (Shld1). Cells were harvested at different times post infection and protein accumulation was analyzed by immunoblot analysis with antibodies recognizing various subunits and isoforms of RNAP II, cellular CTD kinases (cyclin T1, CDK9), or viral proteins (immediate-early protein IE1, early-late protein pUL44, late protein pp71). The protein accumulation of the *dd*FKBP tagged pUL79 was monitored by an antibody recognizing the FKBP-epitope. Representative results from three independent experiments are shown.

To determine whether the pUL79-RNAP II association can stabilize the RNAP II complex to increase its protein levels, we measured RNAP II protein accumulation during HCMV infection in the presence or absence of pUL79 protein. We have previously constructed a recombinant HCMV virus AD*dd*UL79 in which the UL79 coding sequence was tagged with the highly unstable *dd*FKBP domain [42]. This allowed us to abrogate pUL79 function by targeting it for rapid degradation, or maintain its function by stabilizing the protein with the synthetic ligand Shield-1 (Shld1) [42]. Here, we infected HFF cells with AD*dd*UL79 in the presence or absence of Shld1, and analyzed infected cell lysates by immunoblotting at various times post infection. As anticipated, in the presence of Shld1, *dd*FKBP-pUL79 was detected at 72 hpi from total cell lysates (Fig. 3) or nuclear extracts (Fig. S1) using the antibody recognizing the *dd*FKBP epitope. In the absence of Shld1, *dd*FKBP-pUL79 was markedly reduced and barely visible only after prolonged exposure by immunoblot analysis. To confirm this regulation of pUL79 activity, we also examined expression profiles of representative viral immediate-early (IE1), early (pUL44), and late (pp71) proteins. In the presence of pUL79, all three classes of viral proteins were accumulated with the expected kinetics (Fig. 3). In the absence of pUL79, immediate-early and early proteins accumulated normally but the accumulation of the late protein was dramatically reduced (Fig. 3). These results were consistent with the previous study [42], and validated the effectiveness of Shld1-mediated regulation of pUL79 activity in this study. Importantly, the protein levels of Rbp2 and Rpb1 (both total Rpb1 and various CTD-phosphor isoforms) increased as expected when infection progressed [62], [63], but the accumulations were independent of the presence or absence of pUL79 (Fig. 3). Together, these results indicate that total RNAP II as well as its CTD modified forms accumulate during viral infection in a pUL79-independent manner.

### pUL79 alters RNAP II occupancy at viral loci

A previous study showed that MHV-68 ORF30 and ORF34, homologues of HCMV UL91 and UL95, respectively, are required for the recruitment of RNAP II to the viral late promoters [48]. Like ORF30 and ORF34, both UL91 and UL95 were reported to be essential for late gene expression [41], [43]. In this study, we identified pUL95 as a protein partner of pUL79 (Table 1). Therefore, we hypothesized that pUL79 forms a complex with pUL95 and other binding partners to recruit RNAP II and promote assembly of the transcription initiation complex at viral late promoters.

**Figure 4:**
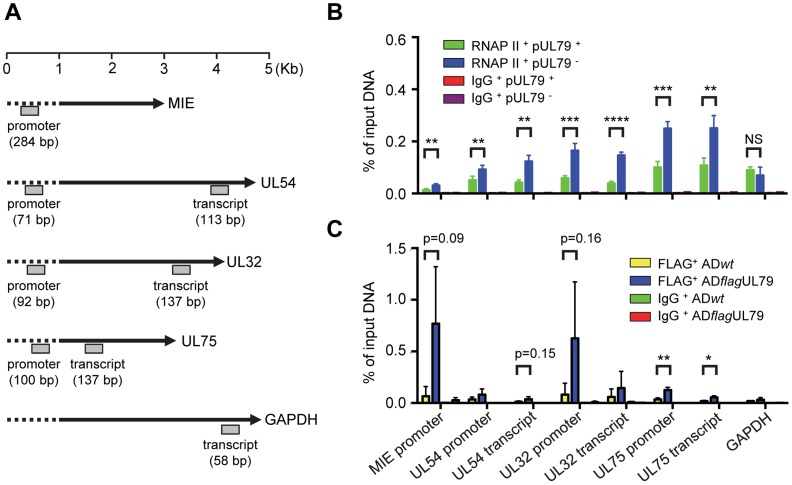
pUL79 alters RNAP II occupancy at viral loci. (A) Schematic representation of the HCMV genes and host GAPDH gene examined by chromatin immunoprecipitation assay (ChIP). Locations and sizes of primer-probe pairs used in ChIP-qPCR analysis are indicated. (B) HFF cells were infected with AD*dd*UL79 at an MOI of 3 in the presence or absence of 1 µM Shld1. Cell extracts were prepared at 72 hpi and analyzed by ChIP assay using rabbit an anti-RNAP II antibody N-20. Normal rabbit IgG was included as a control for non-specific immunoprecipitation. Amounts of input and precipitated (output) DNAs were quantified by qPCR with primers specific for indicated viral loci or human GAPDH. The output-to-input DNA ratios were determined from four independent ChIP experiments with standard deviations calculated by Prism 6 software. Statistical analysis was performed using Student's *t* test (**, P<0.01; ***, P<0.005; ****, P<0.0001; NS, not significant). (C) HFF cells were infected with AD*flag*UL79 or AD*wt* at an MOI of 3. Cell extracts were prepared at 72 hpi and analyzed by ChIP assay using anti-FLAG antibody. Normal mouse IgG was included as a control. Amount of input and precipitated (output) DNAs were quantified by qPCR with primers used in (B). The output-to-input DNA ratios were determined from three independent ChIP experiments with standard deviations calculated by Prism 6 software. Statistical analysis was performed using Student's *t* test (*, P<0.05; **, P<0.01).

To test this, we determined the occupancy of RNAP II on viral late promoters with or without pUL79 during infection using a chromatin immunoprecipitation (ChIP) assay. HFFs were infected with AD*dd*UL79 in the presence or absence of Shld1 and chromatin fractions from infected cells were collected at 72 hpi and analyzed by ChIP assay using a rabbit anti-RNAP II antibody. The amounts of input and output (immunoprecipitated) DNA were measured by quantitative real-time PCR (qPCR) analysis using primers specific to the promoter or transcript regions of viral genes or the cellular housekeeping gene GAPDH (Table S2). The localizations of qPCR primers and sizes of qPCR products are diagramed in Fig. 4A. The qPCR results were presented as relative output-to-input ratios to account for the percentages of host/viral genomes occupied by RNAP II during viral infection (Fig. 4B). The levels of viral and cellular DNA immunoprecipitated by Rbp1 antibody were readily detectable whereas DNA immunoprecipitated by control IgG was minimal, indicating the specific binding of Rbp1 to the DNA sequences detected in this assay. However, to our surprise, the occupancy of Rpb1 at the promoter or transcript regions of viral genes was not reduced in the absence of pUL79, suggesting that pUL79 is not required for RNAP II recruitment to viral promoters (Fig. 4B). Instead, without pUL79, Rpb1 levels on viral DNA were ∼2–2.5 fold higher than those with pUL79. Importantly, during late times of infection (72 hpi), elevated Rpb1 accumulation occurred not only on the loci of viral late genes (UL32 and UL75), it also occurred on those of viral immediate-early genes (MIE) and early genes (UL54) (Fig. 4B). Moreover, this increased association of RNAP II with viral DNA occurred at both promoter regions and transcript regions. By comparison, Rpb1 occupancy on the host gene GAPDH was not altered by pUL79.

If pUL79 modulates RNAP II occupancy on viral loci, we would then expect that pUL79 is associated with RNAP II on viral loci. To test this hypothesis, we determined the occupancy of pUL79 on either viral or host loci during infection. HFFs were infected with AD*flag*UL79 or AD*wt* viruses and chromatin fractions from infected cells were collected at 72 hpi and analyzed by ChIP assay using either an anti-FLAG antibody, which recognizes the 3× FLAG-tagged UL79 protein, or a control IgG antibody. The amounts of input and output (immunoprecipitated) DNA were measured by qPCR analysis using primers identical to those in Fig. 4B. Viral DNA immunoprecipitated by the FLAG antibody from AD*flag*UL79 samples was readily detectable whereas DNA immunoprecipitated by control IgG was minimal, indicating the specific binding of the FLAG tagged pUL79 to the DNA sequences detected in this assay (Figs. 4C and S3). Although certain amounts of background DNA were also immunoprecipitated by the FLAG antibody from AD*wt* samples, the amounts of viral DNA immunoprecipitated from AD*flag*UL79 samples were generally higher, as determined by ChIP-qPCR. This supports the hypothesis that pUL79 occupies viral loci at late times of viral infection. Finally, amounts of cellular DNA (i.e. GAPDH) immunoprecipitated from both AD*flag*UL79 and AD*wt* samples were minimal and indistinguishable, suggesting that pUL79 is not associated with the host genome during viral infection (Fig. 4C).

Taken together, these results indicate that pUL79 regulates the occupancy of RNAP II on viral loci, but not its recruitment to viral promoters, during late times of viral infection.

### pUL79 does not alter a particular phosphorylated form of the RNAP II CTD

**Figure 5:**
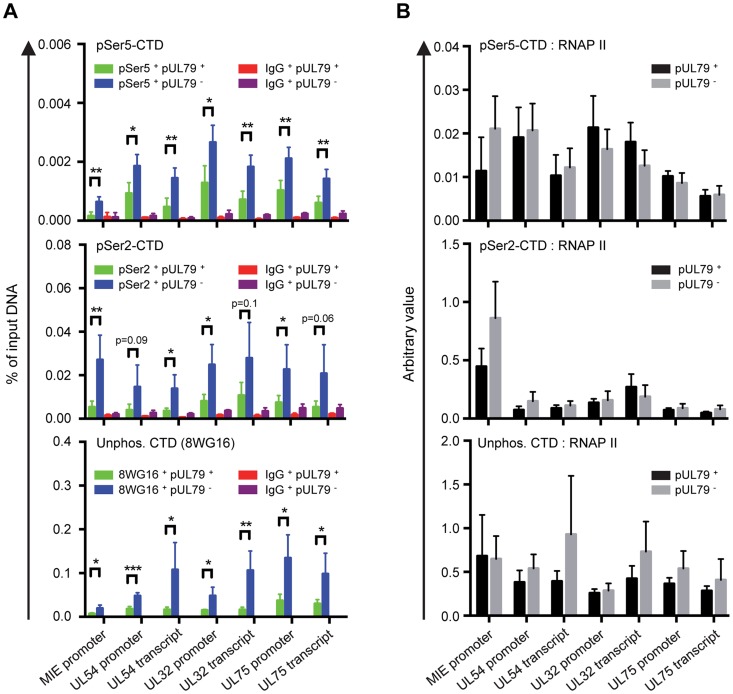
pUL79 does not alter a particular phosphorylated form of the RNAP II CTD domain. HFF cells were infected with AD*dd*UL79 at an MOI of 3 in the presence or absence of 1 µM Shld1. Cell extracts were harvested at 72 hpi and analyzed by ChIP assays. Rabbit antibody to pSer2 CTD, rat antibody to pSer5-CTD, and mouse antibody to non-phosphorylated CTD (8WG16) were used in ChIP assays. Normal rabbit, rat, and mouse IgGs were included as controls for non-specific precipitation, respectively. Immunoprecipitated DNAs were analyzed as described in Fig. 4B and the output-to-input DNA ratios are presented in (A). In addition, the immunoprecipitated amount of each phosphor-isoform of RNAP II CTD relative to that of total RNAP II (immunoprecipitated with antibody N-20) was also calculated and presented in (B). Data from four independent experiments were collected with standard deviations calculated by Prism 6 software. Statistical analysis was performed using Student's *t* test (*, P<0.05; **, P<0.01; ***, P<0.005).

Next, we wanted to determine how the observed dysregulated elevation in the occupancy of RNAP II on viral DNA when pUL79 was abrogated contributed to its diminished ability to transcribe viral genes. Specifically, we wanted to determine which stage of the RNAP II transcription cycle (i.e. initiation, elongation, or termination) was altered by pUL79 by performing ChIP analysis using antibodies that recognize various forms of RNAP II CTD modifications. In a transcription cycle, Ser5 of RNAP II CTD is rapidly phosphorylated (pSer5-CTD) to facilitate the dissociation of RNAP II from the promoter and recruitment of RNA capping and splicing factors. After that, pSer5 CTD levels decrease with a concomitant increase in Ser2 phosphorylation (pSer2-CTD) to facilitate efficient transcription elongation. At 72 hpi, we found that both pSer5-CTD and pSer2-CTD levels significantly increased on viral loci in the absence of pUL79 compared to those in the presence of pUL79 (Fig. 5A). However, the increase of unphosphorlyated CTDs on viral loci also paralleled that of phosphorylated CTD (Fig. 5A). Therefore pUL79 abrogation appeared to elevate all forms of CTD modifications tested at viral loci.

To more specifically determine whether the elevated accumulation of RNAP II on viral DNA arose from a specific CTD modification in the absence of pUL79, we normalized the ChIP occupancy values of pSer5-CTD, pSer2-CTD, and unphosphorylated CTD to that of total RNAP II. Occupancies of various CTD modifications were proportional to that of total RNAP II, and we found no evidence for the preferential occupancy of a particular CTD modification on any viral locus examined (Fig. 5B). Therefore, elevated RNAP II occupancy in the absence of pUL79 was unlikely to be due to the dysregulation of CTD phosphorylation. Consistently, protein levels of CTD kinases (Cyclin T1 and CDK9) and CTD phospho-isoforms (pSer2-CTD, pSer5-CTD, pSer5/pSer2-CTD) were not altered by the presence or absence of pUL79 (Fig. 3). These results together indicate that pUL79 is not involved in phosphorylation of RNAP II CTD, and suggest that without pUL79, RNAP II simply stalls during the transcription cycle, resulting in its elevated accumulation at viral loci.

### pUL79 alters the rate of transcriptional elongation at viral loci

**Figure 6:**
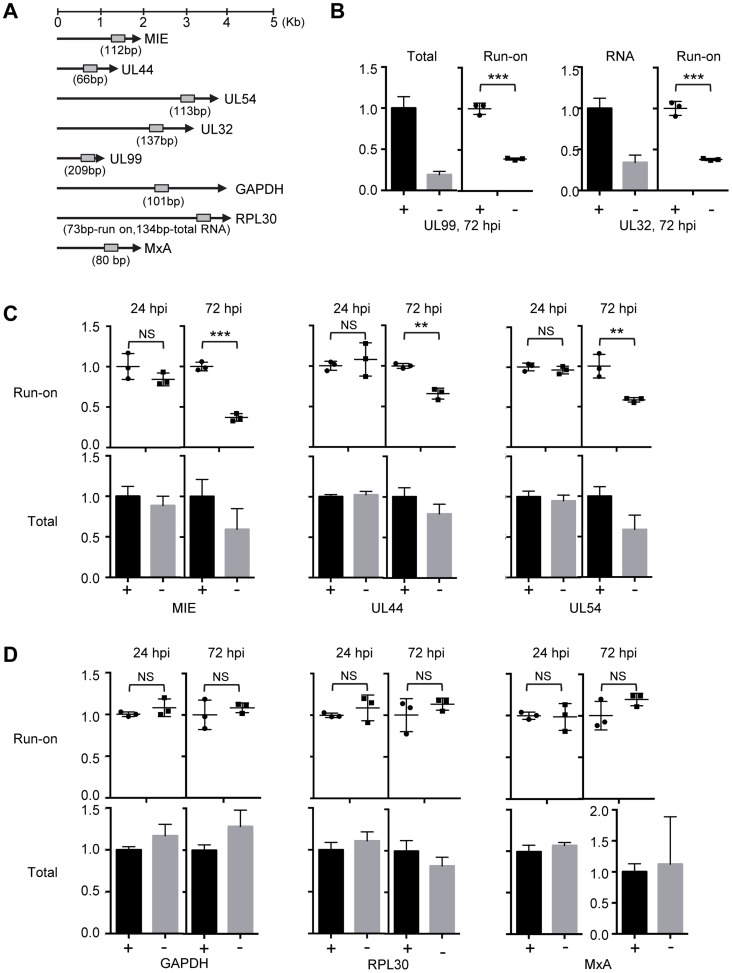
pUL79 alters the rate of transcriptional elongation at viral loci. (A) Schematic representation of the HCMV genes and host genes examined by the nuclear run-on (NRO) assay. Locations and sizes of primer-probe pairs used in subsequent RT-qPCR analysis are indicated. (B–D) HFFs were infected with AD*dd*UL79 at an MOI of 3 in the presence or absence of 1 µM Shld1 (indicated by “+” or “−” sign, respectively). Nuclear extracts were prepared at 24 or 72 hpi and analyzed by NRO assays. Transcription elongation was allowed to resume for 30 minutes in the presence of biotin-labeled UTP, labeled RNA was isolated, and their amounts were determined by RT-qPCR. In addition, accumulations of total RNAs were also determined by RT-qPCR. The normalized amounts of viral run-on transcripts or total transcripts in the presence of Shld1 were set at 1 for the NRO assay or total transcript accumulation analysis, respectively. (B) Relative amounts of total and run-on transcripts of viral late genes UL99 and UL32. (C) Relative amounts of run-on and total transcripts of viral immediate-early and early genes. (D) Relative amounts of run-on and total transcripts of host genes. Data from three independent experiments were collected and standard deviations were calculated by Prism 6 software. Statistical analysis was performed using Student's *t* test (**, P<0.01; ***, P<0.005; NS, not significant).

Based on the above results, we hypothesized that pUL79 was required for efficient elongation of RNAP II-driven transcription at viral loci. To test this, we determined RNAP II elongation activity using a nuclear run-on (NRO) assay. The NRO assay allowed us to monitor the contribution of RNAP II transcriptional activity to transcript levels independent of the effect of RNA stability [70]. To do this, HFF cells were infected with AD*dd*UL79 in the presence or absence of Shld1 and the nuclei of infected cells were isolated at 24 hpi (early timepoint) or 72 hpi (late timepoint) and analyzed by NRO assay. The amounts of newly synthesized run-on RNA were measured by quantitative reverse transcription-coupled quantitative PCR (RT-qPCR) analysis using primers specific to the promoter or transcript regions of viral genes or cellular genes (Fig. 6A and Table S2). Additionally, total RNA was also harvested to monitor the total transcript accumulation.

We found that in the absence of pUL79, the run-on RNA levels of both MIE and late genes (UL99 and UL32) were reduced at late times of infection (72 hpi) to approximately 40% of those in the presence of pUL79 (Figs. 6B–6C). The run-on RNA levels of early genes (UL44 and UL54) without pUL79 were also reduced to approximately 60% of those with pUL79 (Fig. 6C). As RNAP II transcribes at the rate of 1.3–4.0 kb/minute [71], our NRO assay was performed for 30 minutes, which is long enough for RNAP II to transcribe all the viral genes tested. However, without pUL79, RNAP II still failed to transcribe viral genes at the levels comparable to those in pUL79-containing controls at late times of viral infection. We have observed more RNAP II on viral loci in the absence of pUL79 during late stages of viral infection (Figs. 4–5). If these RNAP II complexes functioned properly, we would expect more RNA transcripts to be made in a NRO assay that specifically measured the transcriptional elongation rate. However, we instead found that the RNAP II elongation rate was reduced on viral loci in the absence of pUL79. This is consistent with the hypothesis that the slow-moving RNAP II complexes jammed along viral loci, resulting in its excessive accumulation on viral DNA. Finally, the run-on transcript levels of early genes were indistinguishable at early times of viral infection (i.e. 24 hpi) with or without pUL79 (Fig. 6C). Therefore, we conclude that pUL79 is required for the RNAP II elongation on viral loci at late times of viral infection.

As a control, we also examined the run-on RNA levels of host genes GAPDH, RPL30 (which encodes a 60S ribosomal protein), and MxA (which is a human interferon stimulated gene). Both GAPDH and RPL30 possess a pattern of histone modifications typical of permissive chromatin, similar to those associated with most CMV viral loci during late times of infection [72]. MxA does not encode a TATA box in its promoter [73] and its transcription is suppressed during HCMV infection [74]. In contrast to viral genes, neither the run-on RNA levels nor total RNA accumulations of three host genes were altered by pUL79 at early or late times of viral infection (Fig. 6D). This is consistent with the ChIP analysis in that the occupancy of RNAP II at GAPDH was found unaltered in the absence of pUL79 (Fig. 4B), and indicates that RNAP II does not stall at host genomic loci even without pUL79. Therefore, pUL79 is specifically required for efficient transcription of viral genes but not host genes.

**Figure 7:**
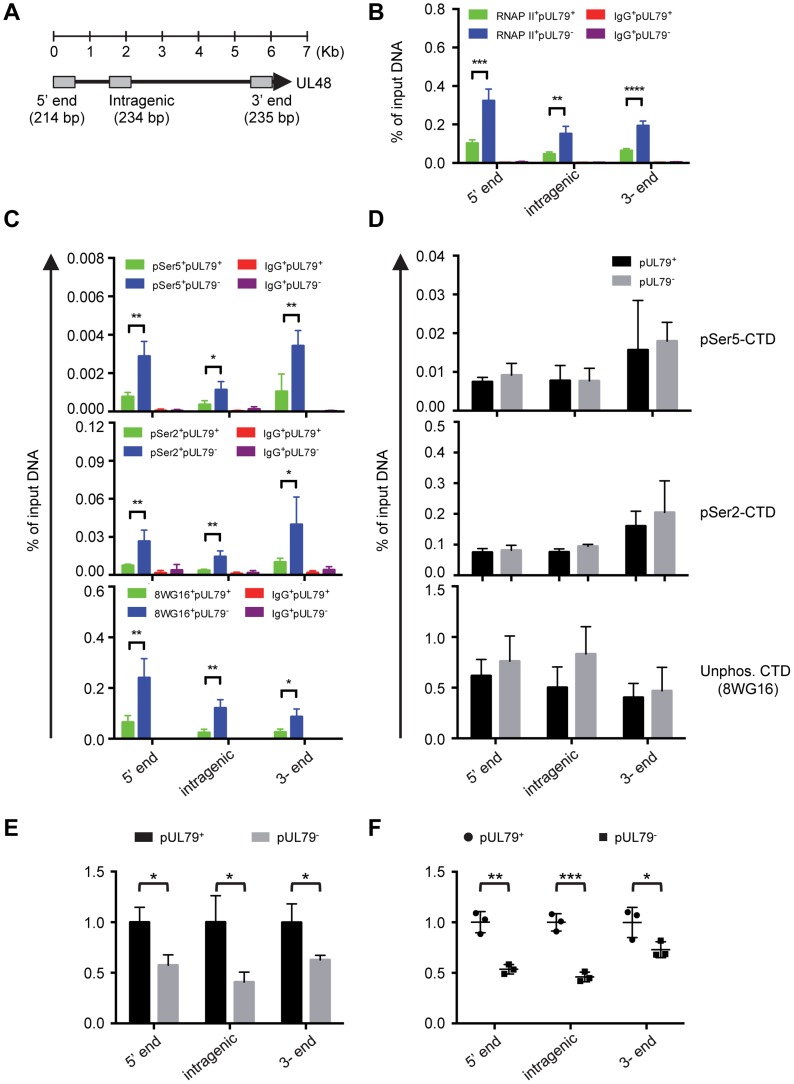
pUL79 alters the rate of transcriptional elongation at various regions of the UL48 gene. (A) Schematic representation of the HCMV gene UL48. Primer-probe pairs used for analysis are indicated. (B–D) HFF cells were infected with AD*dd*UL79 at an MOI of 3 in the presence or absence of 1 µM Shld1. Cell extracts were harvested at 72 hpi and analyzed by ChIP assays as described in Fig. 4 and Fig. 5. (B) represents the output-to-input DNA ratios of RNAP II ChIP. (C) represents the output-to-input DNA ratios of pSer5-CTD, pSer2-CTD, and non-phosphorylated CTD ChIPs. (D) represents the immunoprecipitated amount of each phosphor-isoform of RNAP II CTD relative to that of total RNAP II (immunoprecipitated with antibody N-20). Data from four independent experiments were collected with standard deviations calculated by Prism 6 software. (E–F) HFFs were infected with AD*dd*UL79 at an MOI of 3 in the presence or absence of 1 µM Shld1 (indicated by “pUL79^+^” or “pUL79^−^”, respectively). Nuclear extracts were prepared at 72 hpi and analyzed by NRO assay as described in Fig. 6. Relative amounts of total transcripts and run-on are presented in (E) and (F), respectively. Statistical analysis was performed using Student's *t* test (*, P<0.05; **, P<0.01; ***, P<0.005).

The HCMV genome is dense and many viral regions are transcribed in both directions, resulting in multiple overlapping or co-terminal transcripts. Therefore, the result of analyzing only one viral locus may be complicated by the presence of overlapping transcripts from neighboring genes. We therefore also examined the RNAP II occupancy and elongation rate at multiple loci of UL48 (Fig. 7A), the longest HCMV gene with late kinetics and where RNAP II occupancy has been characterized in a previous study [75]. Similar to other late viral genes that we examined in this study, RNAP II occupancy on all three loci of the UL48 region examined was increased in the absence of pUL79 (Fig. 7B). Without pUL79, RNAP II accumulated excessively throughout the UL48 transcribed region, in proportion to its CTD phosphorylations (Figs. 7C–D). However, UL48 transcripts failed to accumulate efficiently in the absence of pUL79 at late times of viral infection (Fig. 7E). Consistently, at late times of infection RNAP II elongation was reduced on all three UL48 loci in the absence of pUL79, even though the reduction in elongation rates appeared to vary among different UL48 loci (Fig. 7F).

Taken together, our results from the NRO assay provide definitive evidence that pUL79 positively regulates the transcription rates of viral genes but not those of host genes. In the absence of pUL79, RNAP II may still elongate at viral loci but does so at a much slower pace at late times of infection, and ultimately fails to support productive viral gene transcription and viral progeny production.

## Discussion

In this study, we discovered a novel regulatory mechanism of viral transcription mediated by HCMV protein pUL79. We identified cellular RNA polymerase II (RNAP II) as a key factor that interacted with pUL79. This interaction did not alter the overall accumulation of total RNAP II or its various phospho-isoforms during viral infection. Rather, our data suggest that this interaction allowed pUL79 to act as a virus-encoded elongation factor to stimulate transcriptional elongation activity of RNAP II on viral loci during late stages of viral infection where pUL79 is expressed. Without pUL79, RNAP II elongation failed to proceed efficiently and stalled on the viral genome. This caused slow turnover and excessive amounts of RNAP II accumulation on viral loci. Ultimately, this led to the failure of productive viral late transcription and progeny production.

Why is pUL79 only required for viral transcription at late times but not at early times during infection, even though pUL79-mediated regulation occurs at viral loci of all three kinetic classes (immediate-early, early, and late) (Fig. 6)? pUL79 is a late protein and is not expressed until late times of infection. We and others have shown that immediate-early and early genes are transcribed efficiently at early times before pUL79 is expressed (Fig. 6) [42], [43]. It is possible that some transcripts made at early times are stable and persist to late times of infection. When overall transcript accumulations were analyzed, the presence of these pre-existing transcripts could render it difficult to reveal the effect of pUL79 on transcription of immediate-early and early genes at late times during infection. However, the NRO assay measures relative transcription elongation rates at specific gene loci at defined times post infection, and is not affected by pre-existing transcripts. Therefore, it reveals more viral genes than previously expected where pUL79 drives the transcription during late times of viral infection. A more systematic NRO analysis, such as global run-on sequencing (GRO-seq) of virally infected cells, will further define the scope of viral transcription regulated by pUL79.

Many viral factors have been shown to enhance transcription subsequent to initiation through diverse mechanisms. HIV Tat binds to host positive transcription elongation factor (P-TEFb) to remove the blockage of transcription elongation imposed by NELF and DSIF. The Tat/P-TEFb complex stimulates elongation and co-transcriptional processing of proviral transcripts (Reviewed in [76]). During human adenovirus (HAdV) infection, viral protein E1A recruits hPaf1 complex to enhance transcriptional elongation of viral early genes [77]. In herpesviruses, HSV-1 ICP27 interacts with RNAP II CTD to recruit the RNAP II complex to viral promoters [78]. HSV-1 ICP22 binds cdk9 to reduce the serine-2 phosphorylated CTD form of RNAP II [79]–[81]. Together, they regulate the recruitment and proteasome-dependent degradation of RNAP II complex during infection to facilitate viral gene transcription. However, during HCMV infection, RNAP II complex does not undergo extensive protein degradation. In contrast, various isoforms of RNAP II, including the serine-2 phosphorylated CTD form, accumulate as viral infection progresses (Fig. 3). pUL79 does not alter either RNAP II protein accumulation (Fig. 3) or enhance RNAP II recruitment (Fig. 4). Therefore, pUL79 uses a mechanism distinct from other known viral transcriptional elongation regulators to facilitate RNAP II elongation.

Recently, human elongin B was shown to increase the efficiency of RNAP II elongation on viral loci [75]. The siRNA knockdown of elongin B decreases viral mRNA expression as well as reduces RNAP II protein accumulation and occupancy of its serine-2 phosphorylated form on viral loci [75]. Interestingly, elongin B is required for viral mRNA expression of various kinetic classes throughout the whole infection cycle. In contrast, pUL79 is only required at late stages of infection and does not appear to alter the occupancy of various CTD phospho-isoforms of RNAP II on viral loci (Fig. 5). Whether pUL79 interacts with host elongation factors such as elongin B to exert its activity, or how pUL79 selectively modulates the transcription elongation complex at late times of infection, requires further exploration.

**Figure 8:**
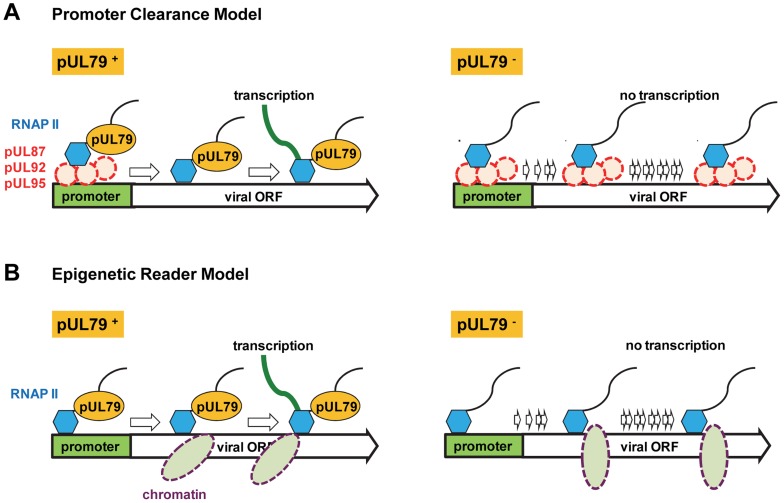
Potential role of pUL79 in RNAP II-mediated viral transcription. During late times of viral infection where pUL79 is expressed, we propose two models where pUL79 may act as an elongation factor to facilitate viral transcription. (A) In the “promoter clearance” model, pUL87, pUL92, pUL95, and potentially other viral factors (shown as red dashed circles) form viral protein pre-initiation complexes (vPIC) to recruit RNAP II to viral promoters. Once transcription initiates, pUL79 interacts with the vPIC to release RNAP II from the vPIC for efficient elongation. In the absence of pUL79, RNAP II is unable to dissociate from vPIC and fails to recruit elongation factors for continued transcription. (B) In the “epigenetic reader” model, pUL79 acts as an epigenetic reader to recognize chromatin modification(s) to facilitate RNAP II elongation. During late times of infection, newly synthesized viral DNA is wrapped with specific histone modifications (shown as purple dashed ovals). pUL79 recognizes these modifications, and then dissociates viral DNA from chromatin binding, with or without other cellular/viral factors, to facilitate RNAP II elongation. In the absence of pUL79, RNAP II is unable to pass through the unopened chromatin, resulting in transcriptional stalling on viral loci.

What is the potential mechanism for pUL79 to modulate the elongation rate of RNAP II? It is possible that pUL79 enhances promoter clearance, a step in which RNAP II transfers from the initiation state to the elongation state (Fig. 8A). During the transcription cycle, RNAP II is recruited to promoters by cellular TATA-box binding protein (TBP) and other general transcription factors (GTFs) to form the pre-initiation complexes (PIC). The PIC places RNAP II at transcription start sites, denatures DNA, and positions DNA into the RNAP II active site for transcription [82]. Once transcription initiates, RNAP II dissociates from the PIC and recruits elongation factors for efficient transcription. The dissociation of RNAP II from the PIC is mediated by TFIIH and other cellular kinases to facilitate exchange between initiation factors and elongation factors [83], [84]. Inefficient dissociation from PIC reduces the rate of RNAP II elongation, resulting in the failure to transcribe genes [83]. Several herpesviral proteins have been reported to act as viral transcription initiation factors to form a unique viral PIC. For example, the homologues of HCMV UL87 in gamma-herpesviruses were reported to encode viral TBPs and regulate late transcript accumulation [23], [50]. However, in general TBP loads onto the promoter independent of other factors, and this is consistent with the observation that EBV BcRF1 (homologue of HCMV pUL87) binds to the viral promoter independent of any other partners [50]. Because of this and also the observation that the total RNAP II accumulation on viral loci is not reduced in the absence of pUL79 (Fig. 4B), we hypothesize that pUL79 is not required for the recruitment of pUL87 or subsequently RNAP II to viral promoters. MHV68 ORF30 and ORF34, homologues of HCMV UL91 and UL95, are shown to be required for RNAP II recruitment to viral late promoters [48]. However, RNAP II recruitment to viral promoters is not reduced in the absence pUL79, suggesting that pUL79 is not required for this putative activity of pUL95 (Fig. 4B). Together, we hypothesize that pUL79 is not required for transcription initiation (Fig. 4B). However, the elongation rate of RNAP II at viral loci is reduced drastically, suggesting that pUL79 is essential for a transcription step downstream of initiation (Fig. 6). Strikingly, pUL79 co-purifies with pUL87 and pUL95, two viral factors potentially involved in viral PIC assembly (Table 1). Therefore, even though pUL79 is unlikely to facilitate pUL87 and pUL95 to mediate viral PIC assembly, it is intriguing to speculate that pUL79 may regulate the activity of pUL87 and pUL95 downstream of transcription initiation. As the viral PIC complex may not be recognized by host dissociation factors, it is possible that pUL79 plays a role in the release of RNAP II from viral PIC prior to elongation (Fig. 8A). To test this, further analysis is required to determine the composition of RNAP II/viral PIC as well as their distribution on the viral DNA.

It is also possible that pUL79 plays a role in epigenetic regulation to modulate viral transcription (Fig. 8B). During HCMV infection, viral DNA is chromatinized and undergoes histone modifications to facilitate gene expression [85]. In particular, upon the onset of viral DNA replication, newly synthesized viral DNA is wrapped with histone 3 with lysine 4 methylation (H3K4me2), a modification that favors active transcription, suggesting the potential involvement of epigenetic regulation in viral late transcription [72]. Even though pUL79 was not required for methylating H3K4 [72], the possibility remains that pUL79 may act as an epigenetic reader to recognize histone modifications unique to viral DNA, and unwrap viral DNA packaged by histones to facilitate RNAP II elongation (Fig. 8B).

How does pUL79 specifically regulate transcription of viral loci? In this study, we showed that pUL79-mediated transcriptional regulation was limited to viral genes, but not host genes (i.e. GAPDH, RPL30, and MxA). This specificity may be partially due to the localization of pUL79 during infection as pUL79 is enriched in viral replication compartments where late viral transcription occurs [42]. In addition, late promoters of beta- and gamma-herpesviruses contain a non-canonical TATA box sequence [50]. EBV BcRF1, the homologue of HCMV pUL87, is a viral TATA-box binding protein which preferentially binds to this non-canonical TATA box over the canonical sequence. This suggests that viral transcription machinery directs RNAP II to viral late promoters during late stages of viral infection [50]. In HCMV, several characterized viral late promoters also contain the same non-canonical TATA sequences [86]–[91]. Therefore, pUL79 may also act as a viral specific TATA-box binding protein. However, in this study we observed an overall decrease in transcription rates among all three kinetic classes of viral loci during late times of infection (Fig. 6). Further analysis is needed to understand how pUL79 can regulate the rate of viral transcription regardless of the structures of gene promoters.

In this study, we found that pUL79 also co-purified with other viral regulators of HCMV late gene expression, suggesting that pUL79 may interact with these regulators to form complexes during viral infection (Table 1). It is not known whether these viral regulators use similar mechanisms to regulate viral transcription. For example, pUL91 and pUL92 were shown to specifically regulate only true late genes [41]. It is possible that these regulators have conserved functions and yet still possess different specificities. In addition, pUL79 also co-purified with viral DNA replication factors (Table 1). Previously, we have shown that pUL79-mediated viral transcription requires the onset of viral DNA synthesis [42]. Expression of neither pUL79 alone nor the combination of all known late gene regulators alters the expression kinetics of viral genes, especially viral late genes [41], [42]. Therefore, it is also possible that viral DNA synthesis events predispose viral DNA to late transcription via interactions between replication factors and pUL79.

In conclusion, we have used a systematic proteomic approach to elucidate the mechanism underlying the activity of the HCMV late gene expression regulator pUL79. pUL79 interacts with RNAP II to modulate its transcription rate at viral loci during late times of viral infection. This unique viral mechanism is potentially conserved among beta- and gamma- herpesviruses, and provides insight into the design of novel antivirals targeting steps after viral DNA synthesis.

## Materials and Methods

### Plasmids and reagents

pYD-C755 (i.e. pLKO) was a pLKO-based lentiviral vector (also referred as pLKO.DCMV.TetO.mcs in [92], a generous gift from Roger Everett, University of Glasgow Centre for Viral Research). pYD-C751 (i.e. pLKO-HA-pUL79) was created by cloning a PCR fragment containing the UL79 coding sequence along with an N-terminal hemagglutinin (HA) tag into the multiple cloning site of pYD-C755. pYD-C744 was derived from pGalK [93], and carried a cassette in which 3×FLAG tag was followed by a GalK/kanamycin dual expression cassette flanked by the Flp recognition target (FRT) sequence [94].

The synthetic chemical ligand Shield-1 (Shld1) used to regulate the stability of *dd*FKBP-tagged proteins was purchased from Cheminpharma (Farmington, CT). Benzonase was purchased from EMD Millipore. The following primary antibodies were used in this study: anti-beta actin (clone AC15, Abcam); anti-FLAG (clone M2/F1804 and M2/F3165, Sigma-Aldrich); anti-HA (clone 16B12, Covance; clone 3F10, Roche); anti FKBP12 (clone 8/FKBP12, BD Biosciences); anti-Rpb1 (clone N-20 from Santa Cruz to detect total Rpb1; or clone 8WG16 from Abcam to detect both total Rpb1 and the unphosphorylated CTD form of Rbp1); anti-Rpb2 (S-20, Santa Cruz); anti-Rpb1 phospho-CTD Ser5/Ser2 (clone H-14, Covance); anti-Rpb1 phospho-CTD Ser5 (clone 3E8, Millipore); anti-Rpb1 phospho-CTD Ser2 (ab5095, Abcam); anti-CDK9 (clone H-169, Santa Cruz); anti-cyclin T1 (clone H-245, Santa Cruz); anti-pUL44 (clone 10D8, Virusys); anti-IE1, anti-pp28, and anti-pp71 (generous gifts from Thomas Shenk, Princeton University).

### Cells and viruses

Primary human newborn foreskin fibroblasts (HFFs) and HEK-293T cells were propagated in Dulbecco modified Eagle medium (DMEM) supplemented with 10% fetal calf serum, nonessential amino acids, sodium pyruvate, and penicillin-streptomycin.

Three HCMV recombinant viruses, AD*wt*, AD*dd*UL79, and AD*flag*UL79, were used in this study. The wildtype virus AD*wt* was reconstituted from the BAC-HCMV clone pAD*wt* (also referred as pAD-GFP in the previous study [42]). pAD*wt* carries the full-length genome of HCMV strain AD169, with the exception that it contains a simian virus 40 (SV40) early promoter-driven green fluorescent protein (GFP) gene in place of the viral US4–US6 region that is dispensable for viral replication in HFFs [95], [96]. AD*dd*UL79 was derived from AD*wt* using BAC recombineering, where the pUL79 coding sequence was fused to that of destabilizing domain *dd*FKBP [42].

AD*flag*UL79 was reconstituted from the BAC clone pAD*flag*UL79. This BAC clone was derived from pAD*wt*, and was constructed by using a linear recombination approach in the bacterial strain SW105 that contained an arabinose-inducible Flp gene for the transient expression of Flp recombinase [94]. Briefly, the cassette that carried 3×FLAG followed by the GalK/kanamycin dual marker was first generated by PCR from pYD-C744 with a pair of 70-bp primers, so that the PCR-generated cassette was also flanked by 50-bp viral sequences immediately upstream or downstream of the 5′-end of the UL79 coding sequence. The cassette was recombined into pAD*wt* at the 5′-end of the UL79 coding sequence by using linear recombination. The GalK/kanamycin marker was subsequently removed by Flp-FRT recombination [94]. The final clone pAD*flag*UL79 contained the 3×FLAG sequence along with a small FRT site fused in frame at the 5′-terminus of the UL79 coding sequence (Fig. 1A).

To reconstitute virus, 2 µg of the BAC-HCMV DNA and 1 µg of the pp71 expression plasmid were transfected into HFF cells by electroporation [96], and the culture medium was changed 24 hours later. For reconstitution of AD*dd*UL79 virus, Shld1 was added every 48 hours to maintain the concentration at 1 µM. Reconstituted virus was harvested by collecting cell-free culture supernatant when the entire monolayer of cells was lysed. To produce virus stocks, cell-free culture supernatants were collected from HFFs infected at an MOI of 0.01. Viruses were pelleted by ultracentrifugation through a 20% D-sorbitol cushion at an average relative centrifugal force of 53,000×g for 1 hour, resuspended in DMEM with 10% tissue fetal calf serum, and saved as viral stocks. HCMV titers were determined by 50% culture infectious dose (TCID_50_) assay in HFFs [42].

### Transient transfection

Four µg of plasmid DNA and 12 µl polyethylenimine (PEI) (1 mg/ml, Polysciences) were mixed with 100 µl OPTI-MEM (Invitrogen) and incubated at room temperature for 10 minutes. The mixture was then added to 900 µl complete medium containing 10% fetal calf serum, and applied to 5×10^6^ HEK-293T cells that were seeded one day before. Cells were incubated for 4 hours before medium was changed.

### Analysis of immunoprecipitation, mass spectrometry, and immunoblotting

For total cell lysates, immunoprecipitation was performed using a protocol modified from previous studies [67], [97], [98]. In brief, HFF cells (5×10^7^) were infected with HCMV AD*flag*UL79 or AD*wt* at a multiplicity of infection (MOI) of 3. At 72 hpi, cells were collected, rinsed twice with cold phosphate-buffered saline (PBS), and lysed in 2 ml EBC2 buffer (50 mM Tris [pH 8.0], 300 mM NaCl, 0.5% NP40) supplemented with protease and phosphatase inhibitors. Cell lysates were then supplemented with 250 unit (U) Benzonase nuclease (Millipore), incubated at 4°C for 15 minutes. One aliquot of cell lysates was saved as the input control and boiled in the LDS sample buffer in the presence of sample reducing agent (Novex). The remainder was clarified by centrifugation at 10,000×g at 4°C for 15 minutes. The supernatant was incubated with protein A-dynabeads (Novex) conjugated with antibody to FLAG (M2) or Rpb1 (N-20) together with an additional 250 U of Benzonase at 4°C overnight. In addition, to confirm the nuclease activity of Benzonase, an aliquot of the supernatant was analyzed on a 0.8% agarose gel containing 100 µg/ml ethidium bromide for the detection of DNA/RNA. The following day the beads were washed three times with 1 ml EBC2 buffer and once with EBC2 buffer without NP40. The immuneprecipitants were eluted by boiling in reducing sample buffer for 5 minutes. For nuclear extracts, immunoprecipitation was performed using the Nuclear Complex Co-IP kit according to the manufacturer's instructions (Active Motif).

For mass spectrometry analysis, cell lysates were prepared in the presence of Benzonase (250 U per 5×10^7^ HFF cells), and the efficiency of enzyme digestion was examined in ethidium bromide-stained agarose gel electrophoresis analysis (Fig. S2). Proteins precipitated by anti-FLAG antibody were resolved on a NuPAGE 4–12% gradient gel (Novex) and subsequently stained using a ProteoSilver Silver Stain kit (Sigma-Aldrich) according to the manufacturer's instruction. Protein bands unique to AD*flag*UL79-infected sample were extracted. In addition, gel bands from the AD*wt*-infected sample with migrating positions corresponding to those of AD*flag*UL79-specific bands were also extracted as negative controls. Extracted gel samples were submitted to the Keck Mass Spectrometry and Proteomics Facility (School of Medicine, Yale University) for liquid chromatography (LC)-mass spectrometry analysis for protein identification.

Protein amounts were determined by immunoblot analysis as previously described [42]. In brief, proteins were resolved on an SDS polyacrylamide gel, transferred to a polyvinylidene difluoride (PVDF) membrane, hybridized with a primary antibody, reacted with the horseradish peroxidase-conjugated secondary antibody, and visualized using chemiluminescent substrate (Thermo Scientific).

### Chromatin immunoprecipitation (ChIP)

The ChIP was performed using the MAGnify chromatin-immunoprecipitation system (Life Technologies) and reagents provided in the kit according to the manufacturer's protocol with modifications. To prepare the chromatin lysates of AD*dd*UL79 infected cells, 2×10^6^ HFFs were infected with AD*dd*UL79 at an MOI of 3.0 in the presence or absence of Shld1. To prepare the chromatin lysates of AD*flag*UL79 or AD*wt* infected cells, 2×10^6^ HFFs were infected with AD*flag*UL79 or AD*wt* viruses without Shld1. At 72 hours, infected cells were washed twice with PBS, trypsinized, and crosslinked with 1% formaldehyde at room temperature with mixing for 10 minutes. Glycine was added to the final concentration of 125 mM and incubated at room temperature for 5 minutes to stop the cross-linking reaction. Cells were collected by centrifugation at 4°C, 200×g for 10 minutes, washed twice in ice-cold PBS, and lysed in 100 µl lysis buffer with protease inhibitors. Chromatins were sheared into 200–500 bp fragments using either a cup-horn Branson Sonifier 450 (30-second pulse and 60% output with 40-second interval for 70 times in ice water) or a NGS Bioruptor (Diagenode) (3×10 cycles of 15-seconds on/45-seconds off in a automatic water cooling system). Samples were gently vortexed every five sonication cycles and allowed to cool in ice water for an additional 2 minutes. Lysates were cleared by centrifugation (20,000×g, 15 minutes; 4°C) and stored as 20-µl aliquots. To confirm the size of sheared chromatin fragments, one 20-µl aliquot was treated with RNase A at 37°C for 1 hour and de-crosslinked by protease K treatment overnight. DNA was purified and analyzed by agarose gel electrophoresis (data not shown).

To immunoprecipitate protein-bound chromatin fragments, each 20-µl aliquot was diluted in dilution buffer with protease inhibitors, and first incubated with 40 µl BSA-preblocked protein A/G Dynabeads to pre-clean for 2 hours. Beads were removed, and one tenth volume of the supernatant was saved as the input sample. The remainder of the supernatant was incubated with appropriate antibodies to generate protein-antibody complexes or with IgG (negative control) (Table S1) at 4°C for 16 hours. Forty µl BSA-preblocked protein A/G Dynabeads (Invitrogen) was added to the samples and incubated at 4°C for another 1.5 hours to immunoprecipitate the complexes. Beads were collected, washed twice with IP Buffer 1 and three times with IP Buffer 2. Protein-antibody complexes were eluted from Dynabeads by incubation with reverse crosslinking buffer with proteinase K at 55°C for 15 minutes. Dynabeads were removed, and crosslinking of protein-antibody complexes in the supernatant were reversed by incubation at 65°C for 15 minutes. In addition, the input sample was also treated with the reverse crosslinking buffer in the same procedure to reverse crosslinking. Both input and immunoprecipitated DNAs were isolated by DNA purification on magnetic beads. DNA fragments were quantified by quantitative PCR (qPCR) using SYBR Select Mix (Invitrogen) kit or Taqman Fast Advanced Master Mix kit (Invitrogen). The sequences of primers and Taqman probes are listed in Table S2.

### Nuclear run-on (NRO) assay

The protocol of the NRO assay was adapted from previous studies with modifications [70], [99], [100]. 1×10^7^ HFFs were infected with AD*dd*UL79 at an MOI of 3 in the presence or absence of Shld1. At 72 hpi, cells were washed twice with PBS, trypsinized, collected by centrifugation (4°C, 270×g), and washed twice with cold PBS again to remove residual calcium and magnesium. To extract nuclei, cell pellets were resuspended in 4 mL cell lysis buffer (10 mM Tris-HCl, pH 7.2, 3 mM MgCl_2_, 10 mM NaCl, 150 mM sucrose, and 0.5% NP40) for 5 minutes on ice. Extracted nuclei were collected by centrifugation (4°C, 170×g) and gently washed with cell lysis buffer to remove NP40. Pellets were resuspended in 300 µl freezing buffer (50 mM Tris-HCl, pH 8.3, 40% glycerol, 5 mM MgCl_2_, and 0.1 mM EDTA), washed once with 1× run-on reaction buffer (20 mM Tris-HCl, pH 7.5, 10 mM MgCl_2_, 150 mM KCl, and 20% (v/v) glycerol). To perform NRO assay, 10^7^ nuclei were incubated in 100 µl 1× run-on reaction buffer with ATP, CTP, GTP (0.5 mM each), and 0.2 mM biotin-16-UTP (Invitrogen) at 29°C for 30 minutes. The reaction was stopped by snap freezing in liquid nitrogen. As negative controls, run-on reactions were also performed with UTP instead of biotin-16-UTP. To isolate biotin-labeled run-on transcripts, streptavidin-coated Dynabeads (Dynabeads MyOne Streptavidin C1, Invitrogen) were resuspended in binding buffer (10 mM Tris-HCl, pH 7.5, 1 mM EDTA, and 2 M NaCl), and mixed with an equal volume of run-on transcripts. The samples were incubated at 42°C for 20 minutes and then at room temperature for 1.5 hours. Beads were collected, and washed twice with 15% formamide and three times with 2× standard saline citrate (Invitrogen). Biotinylated RNAs on the beads were reverse transcribed to generate cDNA using SuperScript VILO cDNA Synthesis Kit (Invitrogen), and quantified by reverse transcription-coupled qPCR (RT-qPCR) analysis. The relative transcript amounts were normalized to those of 18S rRNA (that is transcribed by RNA polymerase I (RNAP I) so is an unbiased internal control for RNAP II activity). In addition, total RNA of infected cells was also isolated separately by TRIzol extraction (Invitrogen) and the amounts were determined by RT-qPCR analysis (see Table S2 for primer sequences).

## Supporting Information

Figure S1 **Accumulation of** ***dd*** **FKBP tagged pUL79 is regulated by Shld1 during infection.** HFFs were infected with AD*dd*UL79 at an MOI of 3 in the presence or absence of 1 µM Shld1. Nuclear extracts were prepared from infected cells at different times post infection, and protein accumulation of the *dd*FKBP tagged pUL79 was monitored by an antibody recognizing the FKBP-epitope. Viral immediate-early protein IE1 and host protein actin were used as infection and loading controls, respectively, and detected by immunoblotting with respective antibodies. Representative results from three independent experiments are shown.
(TIF)

Figure S2 **Nuclease digestion of immunoprecipitated samples from infected cell lysates for mass spectrometry analysis.** To prepare samples for mass spectrometry analysis as depicted in Fig. 1D, cell lysates were treated with or without Benzonase (250 U per 5×10^7^ HFF cells) and the efficiency of enzyme digestion was examined on an ethidium bromide (EtBr)-stained agarose gel. Only Benzonase-treated samples were processed for subsequent mass spectrometry to identify pUL79 protein partners.
(TIF)

Figure S3 **pUL79 is associated with viral loci during HCMV infection.** The data in Fig. 4C are re-graphed with the y-axis scales of the output-to-input DNA ratio proper for each sample set. This allows visualization of the difference between the FLAG-pUL79 samples relative to the untagged pUL79 controls.
(TIF)

Table S1 **Antibodies used in chromatin immunoprecipitation assays.**
(DOCX)

Table S2 **Primers and probes used in ChIP and RT-qPCR analysis.**
(DOCX)
