# Supplementary figures and images for: Human Cytomegalovirus pUL79 Is an Elongation Factor of RNA Polymerase II for Viral Gene Transcription
Source: PLoS Pathog. 2014 Aug 28;10(8):e1004350. doi: 10.1371/journal.ppat.1004350 (PMC4148446; doi:10.1371/journal.ppat.1004350)

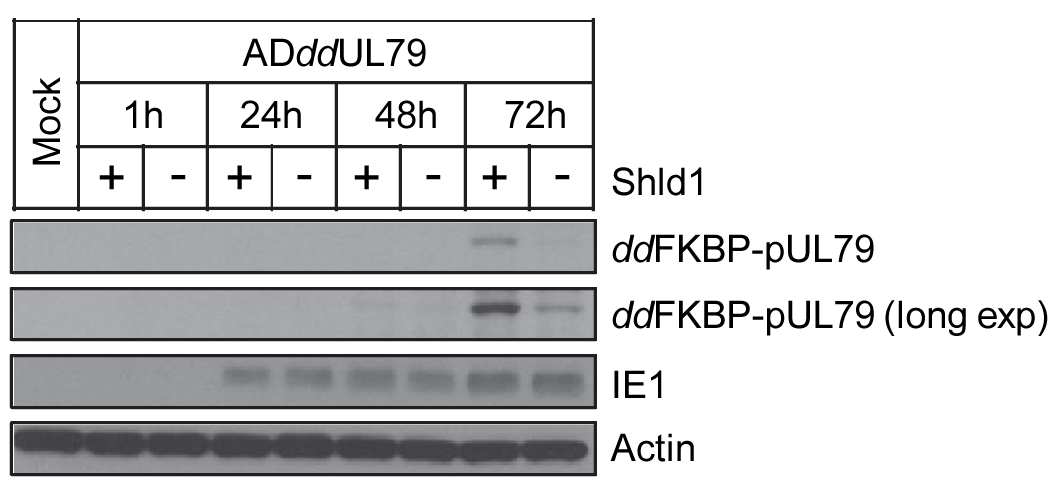

Supplement: Figure S1 — Accumulation of dd FKBP tagged pUL79 is regulated by Shld1 during infection. HFFs were infected with ADddUL79 at an MOI of 3 in the presence or absence of 1 µM Shld1. Nuclear extracts were prepared from infected cells at different times post infection, and protein accumulation of the ddFKBP tagged pUL79 was monitored by an antibody recognizing the FKBP-epitope. Viral immediate-early protein IE1 and host protein actin were used as infection and loading controls, respectively, and detected by immunoblotting with respective antibodies. Representative results from three independent experiments are shown. (TIF) [file ppat.1004350.s001.tif]

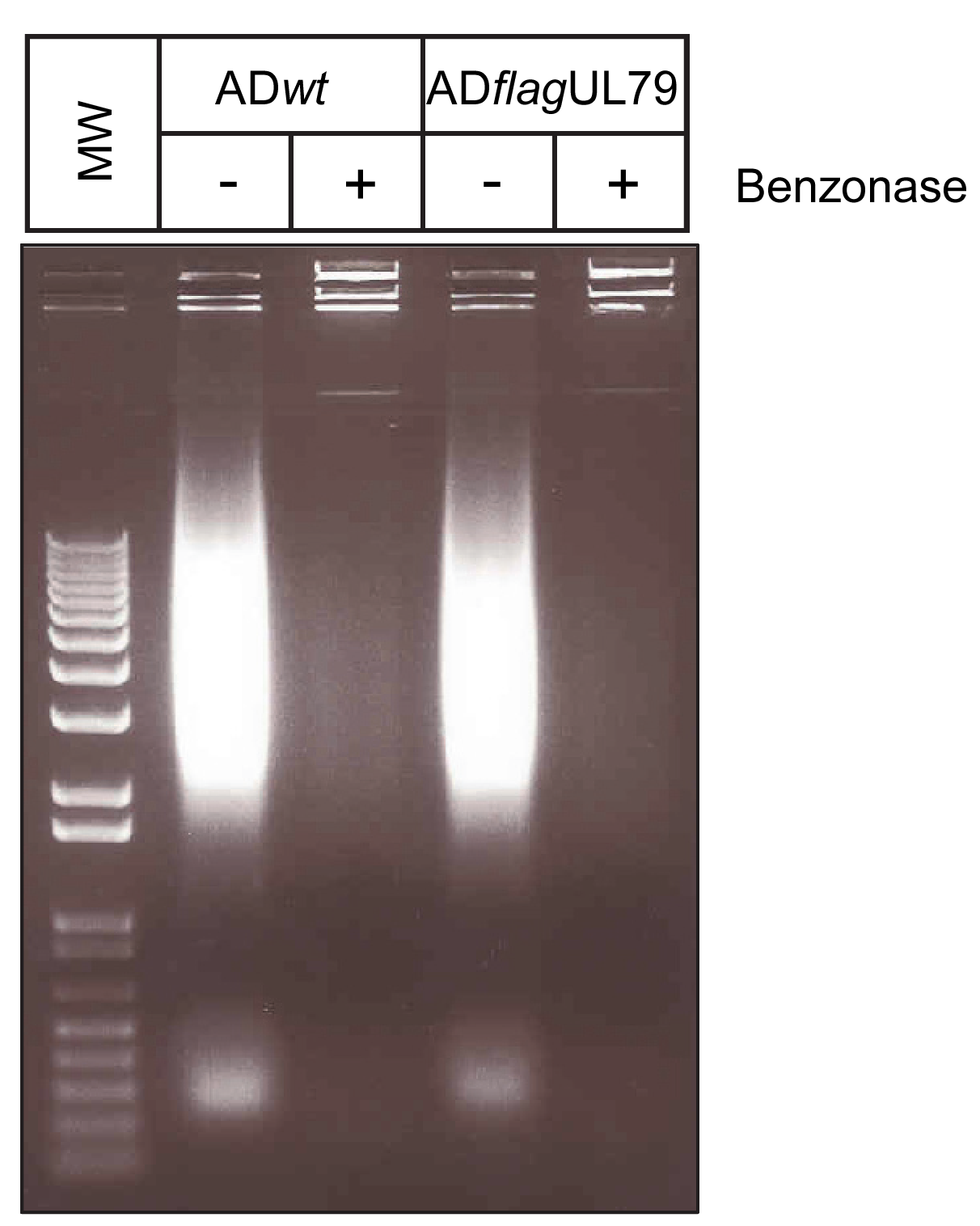

Supplement: Figure S2 — Nuclease digestion of immunoprecipitated samples from infected cell lysates for mass spectrometry analysis. To prepare samples for mass spectrometry analysis as depicted in Fig. 1D, cell lysates were treated with or without Benzonase (250 U per 5×10^7^ HFF cells) and the efficiency of enzyme digestion was examined on an ethidium bromide (EtBr)-stained agarose gel. Only Benzonase-treated samples were processed for subsequent mass spectrometry to identify pUL79 protein partners. (TIF) [file ppat.1004350.s002.tif]

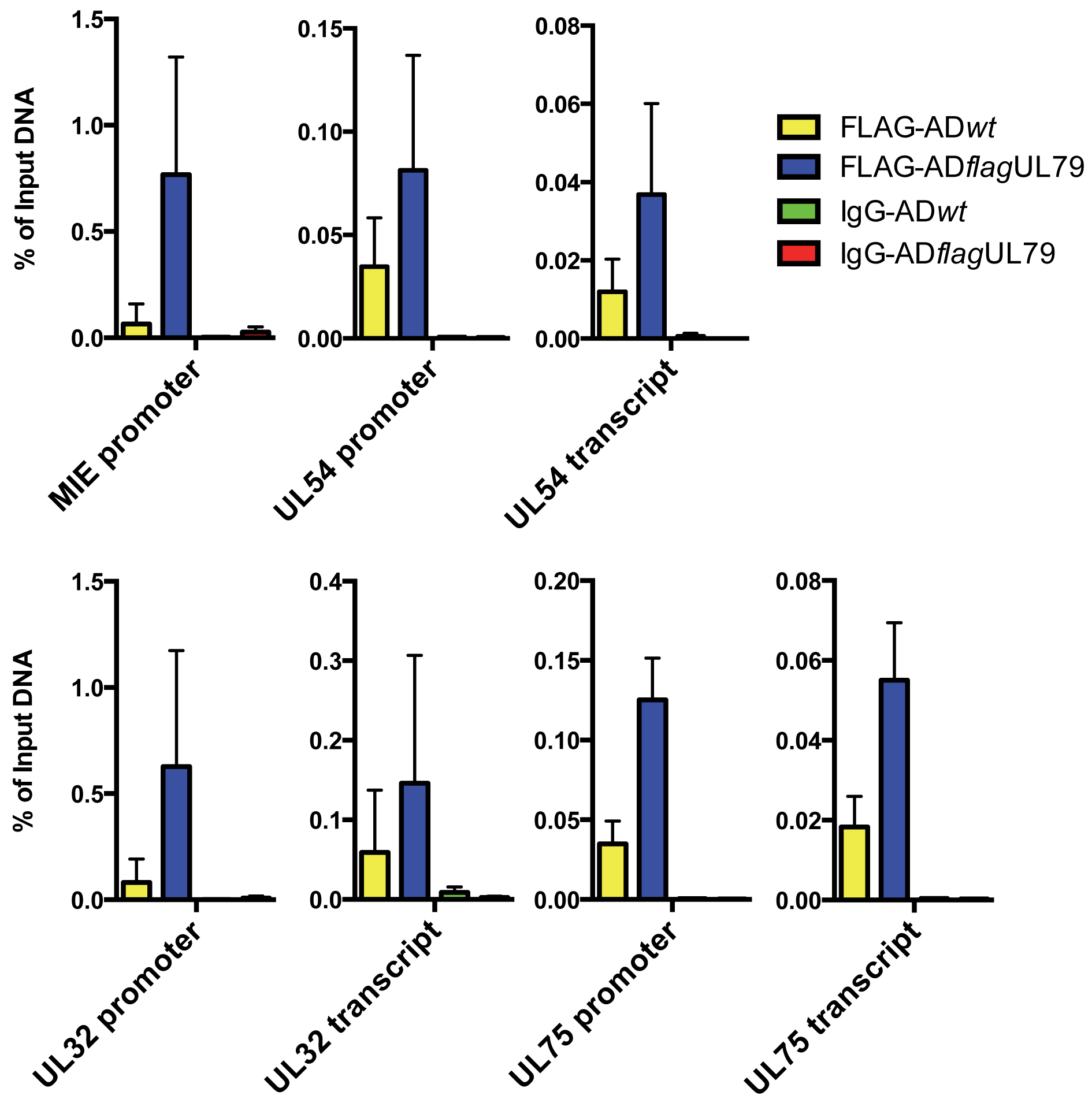

Supplement: Figure S3 — pUL79 is associated with viral loci during HCMV infection. The data in Fig. 4C are re-graphed with the y-axis scales of the output-to-input DNA ratio proper for each sample set. This allows visualization of the difference between the FLAG-pUL79 samples relative to the untagged pUL79 controls. (TIF) [file ppat.1004350.s003.tif]
