# Supplementary material for: Human Cytomegalovirus pUL79 Is an Elongation Factor of RNA Polymerase II for Viral Gene Transcription
Source: PLoS Pathog. 2014 Aug 28;10(8):e1004350. doi: 10.1371/journal.ppat.1004350 (PMC4148446; doi:10.1371/journal.ppat.1004350)
Supplement: Table S1 — Antibodies used in chromatin immunoprecipitation assays. (DOCX) [file ppat.1004350.s004.docx]

**Table S1. Antibodies used in chromatin immunoprecipitation assays**

| Antigen | Clone No | Source | Type | Amount*^a b^* | Dynabead*^c^* |
| --- | --- | --- | --- | --- | --- |
| FLAG | M2/F3165 | Sigma | Mouse monoclonal | 6 μg | Protein A/G |
| Normal mouse IgG  (negative control) | 100005292 | Invitrogen | Mouse polyclonal | 2.5-6 μg | Protein A/G |
| Normal rabbit IgG  (negative control) | 100005291 | Invitrogen | Rabbit polyclonal | 1 μg | Protein A/G |
| Normal rat IgG  (negative control) | NI04 | Millipore | Rat polyclonal | 5 μg | Protein G |
| RNAP II, CTD phosphorylated Ser2 (pSer2) | ab5095 | Abcam | Rabbit polyclonal | 1 μg | Protein A/G |
| RNAP II, CTD phosphorylated Ser5 (pSer5) | 3E8 | Millipore | Rat monoclonal | 5 μg | Protein G |
| RNAP II  unphosphorylated CTD | 8WG16 | Abcam | Mouse monoclonal | 2.5 μg | Protein A/G |
| RNAP II, total | N-20 | Santa Cruz | Rabbit polyclonal | 1 μg | Protein A/G |

*^a^* Amount of antibody used in a 2.5×10^5^-cell sample.

*^b^* 2.5 μg and 6 μg of normal mouse IgG were used as the negative control for mouse 8WG16 and FLAG antibodies, respectively.

*^c^* Type of Fc-binding proteins conjugated to Dynabeads.
