# Supplementary material for: Human Cytomegalovirus pUL79 Is an Elongation Factor of RNA Polymerase II for Viral Gene Transcription
Source: PLoS Pathog. 2014 Aug 28;10(8):e1004350. doi: 10.1371/journal.ppat.1004350 (PMC4148446; doi:10.1371/journal.ppat.1004350)
Supplement: Table S2 — Primers and probes used in ChIP and RT-qPCR analysis. (DOCX) [file ppat.1004350.s005.docx]

**Table S2. Primers and probes used in ChIP and RT-qPCR analysis**

| Primer ID | Sequence (5' - 3') | Target | Reference |
| --- | --- | --- | --- |
| YD-Pri2527 | CCACGATGGACACCATCATC | UL32, forward *^a^* |  |
| YD-Pri2528 | AGAAAACCGCGTATCCGCCT | UL32, reverse *^a^* |  |
| YD-Pri2074 | GGTTTCTGGCTCGTGGATGTCG | UL32, forward | [[1](#_ENREF_1)] |
| YD-Pri2075 | CACACAACACCGTCGTCCGATTAC | UL32, reverse | [[1](#_ENREF_1)] |
| YD-Pri692 | TTTTCTCACCGAGGAACCTTTC | UL44, forward | [[2](#_ENREF_2)] |
| YD-Pri693 | CCGCTGTTCCCGACGTAAT | UL44, reverse | [[2](#_ENREF_2)] |
| YD-Pri2076 | GTGTCCCATTCCCGACTCG | UL99, forward | [[1](#_ENREF_1)] |
| YD-Pri2077 | TTCACAACGTCCACCCACC | UL99, reverse | [[1](#_ENREF_1)] |
| YD-Pri2489 | CTTACGGGACTTTCCTACTTG | MIE, forward *^a^* | [[3](#_ENREF_3)] |
| YD-Pri2490 | CGATCTGACGGTTCACTAA | MIE, reverse *^a^* | [[3](#_ENREF_3)] |
| YD-Pri2491 | CCTAGTGTGGATGACCTA | MIE, forward | [[3](#_ENREF_3)] |
| YD-Pri2492 | GTGACACCAGAGAATCAG | MIE, reverse | [[3](#_ENREF_3)] |
| YD-Pri2493 | CACCAAAGACACGTCGTT | UL54, forward *^a^* | [[3](#_ENREF_3)] |
| YD-Pri2494 | GTCCTTTGCGACCAGAAT | UL54, reverse *^a^* | [[3](#_ENREF_3)] |
| YD-Pri2495 | GTGTGCAACTACGAGGTA | UL54, forward | [[3](#_ENREF_3)] |
| YD-Pri2496 | GACAGCACGTTGGTTACA | UL54, reverse | [[3](#_ENREF_3)] |
| YD-Pri2511 | GGGTGTGGCGCTACGGGTTACAAA | UL75, forward *^a^* |  |
| YD-Pri2512 | ATGGCTTACCCGCGTGTCCC | UL75, reverse *^a^* |  |
| YD-Pri2521 | GTGTCCATCAAAGAGGATACAG | UL75, forward |  |
| YD-Pri2522 | AAACCACTCCACACGACTGG | UL75, reverse |  |
| YD-Pri3043 | ATGAAAGTCACACAGGCCAGC | UL48 (5’ end), forward | [[4](#_ENREF_4)] |
| YD-Pri3044 | GCAGCTTCTTTTGCAACTCGC | UL48 (5’ end), reverse | [[4](#_ENREF_4)] |
| YD-Pri3039 | CAACGTTTCGTAACCAAGCGA | UL48 (intragenic), forward | [[4](#_ENREF_4)] |
| YD-Pri3040 | CTGCAGCGCTTTCAGAATTTC | UL48 (intragenic), reverse | [[4](#_ENREF_4)] |
| YD-Pri3041 | CTGCTCCAGGACACGTGGAC | UL48 (3’ end), forward | [[4](#_ENREF_4)] |
| YD-Pri3042 | GGTCATACAGCGGGAAGGTG | UL48 (3’ end), reverse | [[4](#_ENREF_4)] |
| YD-Pri3021 | GTAACCCGTTGAACCCCATT | 18s rRNA, forward |  |
| YD-Pri3022 | CCATCCAATCGGTAGTAGCG | 18s rRNA, reverse |  |
| YD-Pri744 | CTGTTGCTGTAGCCAAATTCGT | GAPDH, forward | [[1](#_ENREF_1)] |
| YD-Pri745 | ACCCACTCCTCCACCTTTGAC | GAPDH, reverse | [[1](#_ENREF_1)] |
| YD-Pri3023 | CAAGGCAAAGCGAAATTGGT | RPL30, forward *^b^* | [[5](#_ENREF_5)] |
| YD-Pri3024 | GCCCGTTCAGTCTCTTCGATT | RPL30, reverse *^b^* | [[5](#_ENREF_5)] |
| YD-Pri3027 | CTGGGCAGTTGTTAGCGAGA | RPL30, forward *^c^* | [[5](#_ENREF_5)] |
| YD-Pri3028 | GTCGCTGGAGTCGATCAACT | RPL30, reverse *^c^* | [[5](#_ENREF_5)] |
| YD-Pri3047 | TCTTCATGCTCCAGACGTAC | MxA, forward | [[5](#_ENREF_5)] |
| YD-Pri3048 | CCAGCTGTAGGTGTCCTTG | MxA, reverse | [[5](#_ENREF_5)] |
| YD-Pri3044 | FAM-AATCAATGCGTCTGCAACGGC  ATCA-TAMRA (Taqman probe, IDT) | UL48 (5’ end) | [[4](#_ENREF_4)] |
| YD-Pri3045 | FAM-ACTTTCAGCGTCTGACGCAGG  TCAT-TAMRA (Taqman probe, IDT) | UL48 (intragenic) | [[4](#_ENREF_4)] |
| YD-Pri3046 | FAM-ACGGCTACGCGATTACCTGCG  TTTC-TAMRA (Taqman probe, IDT) | UL48 (3’ end) | [[4](#_ENREF_4)] |
| Hs03929097_g1 | Taqman probe, Invitrogen | GAPDH |  |

*^a^* Promoter regions

*^b^* Used in RT-qPCR for nuclear run-on assay only

*^c^* Used in RT-qPCR for total transcript quantification

**References**

1. Perng YC, Qian Z, Fehr AR, Xuan B, Yu D (2011) The human cytomegalovirus gene UL79 is required for the accumulation of late viral transcripts. J Virol 85: 4841-4852.

2. Isomura H, Stinski MF, Kudoh A, Nakayama S, Iwahori S, et al. (2007) The late promoter of the human cytomegalovirus viral DNA polymerase processivity factor has an impact on delayed early and late viral gene products but not on viral DNA synthesis. J Virol 81: 6197-6206.

3. Nitzsche A, Paulus C, Nevels M (2008) Temporal dynamics of cytomegalovirus chromatin assembly in productively infected human cells. J Virol 82: 11167-11180.

4. Hwang J, Saffert RT, Kalejta RF (2011) Elongin B-mediated epigenetic alteration of viral chromatin correlates with efficient human cytomegalovirus gene expression and replication. MBio 2: e00023-00011.

5. Knoblach T, Grandel B, Seiler J, Nevels M, Paulus C (2011) Human cytomegalovirus IE1 protein elicits a type II interferon-like host cell response that depends on activated STAT1 but not interferon-gamma. PLoS Pathog 7: e1002016.
